# Nonannual seasonality of influenza‐like illness in a tropical urban setting

**DOI:** 10.1111/irv.12595

**Published:** 2018-08-21

**Authors:** Ha Minh Lam, Amy Wesolowski, Nguyen Thanh Hung, Tran Dang Nguyen, Nguyen Thi Duy Nhat, Stacy Todd, Dao Nguyen Vinh, Nguyen Ha Thao Vy, Tran Thi Nhu Thao, Nguyen Thi Le Thanh, Phan Tri Tin, Ngo Ngoc Quang Minh, Juliet E. Bryant, Caroline O. Buckee, Tran Van Ngoc, Nguyen Van Vinh Chau, Guy E. Thwaites, Jeremy Farrar, Dong Thi Hoai Tam, Ha Vinh, Maciej F. Boni

**Affiliations:** ^1^ Oxford University Clinical Research Unit Wellcome Trust Major Overseas Programme Ho Chi Minh City Vietnam; ^2^ Center for Communicable Disease Dynamics Department of Epidemiology Harvard T.H. Chan School of Public Health Boston Massachusetts; ^3^ Department of Ecology and Evolutionary Biology Princeton University Princeton New Jersey; ^4^ Liverpool School of Tropical Medicine Liverpool UK; ^5^ Viet My Clinics Ho Chi Minh City Vietnam; ^6^ Children's Hospital No. 1 Ho Chi Minh City Vietnam; ^7^ Centre for Tropical Medicine and Global Health Nuffield Department of Medicine University of Oxford Oxford UK; ^8^ Hospital for Tropical Diseases Ho Chi Minh City Vietnam; ^9^ Wellcome Trust London UK; ^10^ Department of Infectious Diseases Pham Ngoc Thach University of Medicine Ho Chi Minh City Vietnam; ^11^ Center for Infectious Disease Dynamics Department of Biology Pennsylvania State University University Park Pennsylvania

## Abstract

**Background:**

In temperate and subtropical climates, respiratory diseases exhibit seasonal peaks in winter. In the tropics, with no winter, peak timings are irregular.

**Methods:**

To obtain a detailed picture of influenza‐like illness (ILI) patterns in the tropics, we established an mHealth study in community clinics in Ho Chi Minh City (HCMC). During 2009‐2015, clinics reported daily case numbers via SMS, with a subset performing molecular diagnostics for influenza virus. This real‐time epidemiology network absorbs 6000 ILI reports annually, one or two orders of magnitude more than typical surveillance systems. A real‐time online ILI indicator was developed to inform clinicians of the daily ILI activity in HCMC.

**Results:**

From August 2009 to December 2015, 63 clinics were enrolled and 36 920 SMS reports were received, covering approximately 1.7M outpatient visits. Approximately 10.6% of outpatients met the ILI case definition. ILI activity in HCMC exhibited strong nonannual dynamics with a dominant periodicity of 206 days. This was confirmed by time series decomposition, stepwise regression, and a forecasting exercise showing that median forecasting errors are 30%‐40% lower when using a 206‐day cycle. In ILI patients from whom nasopharyngeal swabs were taken, 31.2% were positive for influenza. There was no correlation between the ILI time series and the time series of influenza, influenza A, or influenza B (all *P *>* *0.15).

**Conclusion:**

This suggests, for the first time, that a nonannual cycle may be an essential driver of respiratory disease dynamics in the tropics. An immunological interference hypothesis is discussed as a potential underlying mechanism.

## 1 INTRODUCTION

One of the most important challenges facing big‐data studies in all fields is that the larger the data set the less precisely targeted each data point is in answering a specific question. Nowhere is this more apparent than in the big‐data approaches used in infectious disease surveillance, where the volume of data has allowed many types of associations to be investigated,^1^, ^2^, ^3^, ^4^, ^5^, ^6^ but the distance between the source data (an online search, a news story, a social media post) and the presupposed condition (infection with a pathogen) is large enough to warrant additional inquiry into the validity of the association. Indeed, this has been done by several research groups for Google's flu prediction algorithm Google Flu Trends.^7^, ^8^, ^9^, ^10^ Critiques of the algorithm included its reliance on Internet search behavior remaining constant, an overfitting effect that may have given too much weight to associations that were present in training data sets only, as well as specific examples of incorrect forecasts.^9^, ^11^ The challenge in big‐data disease surveillance is to narrow the gap between the infection and the data point describing it and to find a way to generate large data sets where the data points are grounded in the presence of virus, genetic material, an antibody profile, or a set of symptoms. This study presents an attempt at narrowing this gap, and like some of the early big‐data studies^1^, ^12^, ^13^, ^14^, ^15^ is focused on respiratory disease and influenza virus.

In temperate countries, influenza virus is one of the most studied disease systems, exhibiting a predictable wintertime transmission season and a robust relationship between syndromic and molecular surveillance. Little is known about the epidemiology of influenza virus in the tropics despite a renewed research interest in tropical influenza over the past decade resulting from increased availability of influenza surveillance and sequence data.^16^, ^17^, ^18^, ^19^, ^20^ To date, research on tropical influenza has concentrated on whether influenza epidemics exhibit annual seasonality^21^, ^22^, ^23^, ^24^, ^25^, ^26^, ^27^, ^28^, ^29^ and whether influenza viruses show patterns of year‐round persistence.^30^, ^31^, ^32^, ^33^, ^34^ A third question that has received less attention is whether syndromic influenza‐like illness (ILI) surveillance has the same peaks and troughs as molecular surveillance for influenza virus in these regions. In temperate countries, public health agencies are able to rely on ILI reporting to signal the onset of the influenza season,^1^, ^35^, ^36^ but it is not known whether ILI and influenza correlate in tropical countries.^37^, ^38^

The majority of epidemiological studies looking at influenza and/or respiratory disease in the tropics have two major drawbacks. The first is ignoring absolute case counts and reporting only the percentage of samples (nose/throat swabs) that test positive for influenza.^26^, ^29^, ^38^, ^39^, ^40^, ^41^ Ignoring case counts makes it impossible to determine whether samples are being taken during an influenza season or outside of it. The second drawback is underpowering the analysis using a short time series or monthly data or both.^37^, ^38^, ^39^, ^40^, ^42^, ^43^, ^44^, ^45^, ^46^ Monthly data are normally too coarse to infer the presence of an annual transmission season or other periodic trends (if these exist) unless the time series is very long. In fact, this is one of the reasons for disagreement in the current literature as some studies on respiratory disease in the tropics claim support for an annual transmission season^21^, ^26^, ^29^, ^39^, ^40^, ^42^, ^47^, ^48^, ^49^ while others show mixed or no evidence.^22^, ^27^, ^46^, ^50^, ^51^, ^52^, ^53^, ^54^ Among these, some of the more weakly supported results are being used in public health policy to advocate for particular vaccination timings based on incorrectly identified seasonal signals.^29^, ^49^ For influenza virus specifically, studies with sufficient data^27^, ^28^, ^55^ have generally found that annual seasonal signals are not supported in the tropics.

Understanding the dynamics of respiratory disease and influenza in the tropics—especially the presence or absence of annual seasonality—may allow the forecasting methods currently deployed in temperate countries^56^, ^57^, ^58^, ^59^ to be used for tropical influenza. Current forecasting methods rely on mechanistic susceptible‐infected‐recovered (SIR) models and known/inferred climate associations to accurately predict increases in influenza virus infections. In the tropics, it is not known whether influenza dynamics obey classic SIR models, whether they are characterized by low‐level persistence, or a combination of the two. It is also not known which climate‐influenza associations are expected to be present in tropical countries despite accumulating evidence that absolute humidity may be the most influential climate factor.^28^, ^60^ Essentially, the absence of winter in tropical countries makes respiratory disease forecasting much more difficult than in temperate or subtropical climates. If the intrinsic epidemiological dynamics and the presence/absence of climate associations can be understood in the tropics, forecasting of influenza epidemics may be possible. Thus far, the only attempt at influenza forecasting for the subtropics reported that the majority of forecast attempts (lead time >2 weeks before epidemic peak or onset) had accuracies below 50% when predicting the timing, onset, magnitude, or duration of an influenza epidemic,^61^ and no forecasts have been developed for tropical regions.

An accurate description of the basic epidemiology of tropical influenza is critical for inferring the likely routes of viral seeding from the tropics to temperate zones and vice versa.^17^, ^62^ Although there is abundant phylogeographic evidence linking tropical and temperate influenza sequences,^20^ very few analyses have investigated the epidemiological characteristics of tropical influenza and how these affect epidemics in temperate zones. Two exceptions can be seen in Brazil and China, both of which span multiple climatic zones. In Brazil, a pneumonia and influenza mortality time series dating back to 1979 shows an annual influenza epidemic progressing from tropical to temperate parts of Brazil.^30^ A second example can be seen in a study published using sentinel surveillance data from in China, showing the transition from large wintertime influenza peaks in the north to smaller less predictable peaks in the subtropics.^63^ Beyond these two examples, epidemiological links between the tropics and other regions are hard to demonstrate due to the paucity of long‐term consistent surveillance data in tropical regions.

To investigate the fine‐scale epidemiology of respiratory disease dynamics in the tropics and evaluate the potential for forecasting, in August 2009, we set up a real‐time community‐based participatory epidemiology network in Ho Chi Minh City, Vietnam. Our hypothesis was that ILI trends in Ho Chi Minh City would not be annual. Enrolled outpatient clinics across the city reported daily case numbers of ILI by standard mobile phone SMS messages. A subset of the clinics provided molecular confirmations of influenza virus to assess the relationship between ILI and influenza. Our goals were to make daily reporting of ILI as simple as possible in order to encourage frequent reporting and wide participation and to create a real‐time ILI surveillance system that could be used by health professionals in Ho Chi Minh City. Our study is most similar to the clinic‐centered mHealth system setup in Senegal^45^ and Madagascar,^64^ and the benefits of this type of real‐time, big‐data epidemiology can be seen in the dengue hotline system recently described by Rehman et al.^65^ The purpose of our study was to build a long‐term consistent time series of both ILI reports and influenza molecular confirmations. We analyzed the data with traditional time series decomposition to detect periodic signals, with stepwise regression analyses to determine the importance of climate and other covariates, and with regression‐based forecasting to determine the predictability of ILI trends in Ho Chi Minh City.

## 2 MATERIALS AND METHODS

### 2.1 ILI data

In August 2009, a participatory epidemiology study was established in Ho Chi Minh City, Vietnam, in collaboration with the Hospital for Tropical Diseases in Ho Chi Minh City (HCMC) and with permission from the Ho Chi Minh City Department of Health. Participating outpatient clinics report the daily number of total patients seen, the daily number of patients meeting the European CDC definition of ILI,^66^ and the number of hours each clinic was open. To meet the ECDC definition of ILI, a patient must present with (a) sudden onset of symptoms within the past 3 or 4 days; (b) one or more of the following general symptoms (i) fever with axillary temperature above 37.5°C, (ii) malaise, (iii) headache, and (iv) myalgia; and (c) one or more of the following respiratory symptoms (i) cough, (ii) rhinorrhea, (iii) sore throat, and (iv) dyspnea. To encourage enrollment and reduce dropout, clinics are advised to send daily reports by standard mobile phone short messaging system (SMS) text messages; reporting with log books and email is also available. SMS messages are automatically passed to a text‐parsing and data‐cleaning system that was set up and is still actively managed by the Oxford University Clinical Research Unit (OUCRU) in HCMC. Every day, ILI reports are manually approved by a qualified project team member at OUCRU; on approval, they are automatically entered into a mySQL database that holds all data points for the study. A small number of clinics (about 8%) did not use SMS reporting (by their request) and instead emailed ILI numbers to the project team or wrote them down in a daily logbook provided by OUCRU. As part of the data processing pipeline, reports by email or logbook were regularly merged into the main mySQL database. There was no obviously apparent difference in ILI numbers when comparing clinics that used SMS, email, and logbook reporting.

Community engagement meetings were run for the first several years of the study to distribute and explain the study protocol, and a basic leaflet outlining the goals of the study and the reporting methodology was distributed to interested physicians. All documents were translated into Vietnamese, and annual reports and ILI trends were fed back to the clinics on a regular basis. A total of 63 clinics were enrolled in the initial study period (August 2009‐December 2015). Clinics that reported frequent zeros (>50%), or withdrew too early (contributed <200 reports), were not considered for the analysis. The clinics included mostly single‐doctor clinics, some that were open early morning and late evening only (to accommodate a full‐time working schedule for that doctor at a city hospital) and some that were open day‐time hours as that clinician's primary source of income. A few of the clinics were larger polyclinics with several doctors (three to five) and several nurses (five to ten) on staff, a waiting area, one or two patient beds for day‐time only inpatient stay, and the ability to see between 100 and 200 patients per day. The presenting symptoms for patients attending the clinics in this study included ILI, fever, rash, skin infections, nausea, diarrhea, dehydration, conjunctivitis, muscle ache, joint pain, and physical cuts/scrapes/injuries from motorbike (or other) accidents.

In May 2012, a new study component was launched for 24 clinics that agreed to periodic collection of nasopharyngeal (NP) swabs so that a subset of ILI patients could be molecularly confirmed as positive or negative for influenza virus. A swabbing schedule was made at random every year, so that each clinic would be visited an approximately equal number of times, with two clinics selected for swabbing each week. In other words, each clinic was visited two or three times per year, and each week (excepting holidays and the early months of the swabbing substudy), there were two clinic visits lasting 3 days each; the schedule was designed in this way so that no single clinic would have too many visits, as some doctors viewed these as disruptive to the clinic's normal patient flow. Numbers of NP swabs collected each week depended on the numbers of ILI cases presenting at the clinics as well as patient consent.

The research protocol was approved by the Oxford Tropical Research Ethics Committee at the University of Oxford and by the Scientific and Ethical Committee of the Hospital for Tropical Diseases in Ho Chi Minh City.

### 2.2 Molecular confirmation

Respiratory specimens (nasal/throat swabs) were collected from ILI patients at outpatient clinics, transported the same day on ice to OUCRU, and stored in −80°C freezers for a maximum of 3 months before RNA extraction and influenza A and B PCR testing. All specimens were tested by real‐time PCR using primers, probes, and reagents recommended by the World Health Organization (WHO) and the Centers for Disease Control and Prevention (CDC). Sequences of probes and primers used can be referred to in Table S1.

Viral RNA was extracted from 140 μL of a patient's specimen to attain a final elution volume of 50 μL. The extraction was carried out using a MagNA Pure 96 automated system (Roche Applied Science, Penzberg, Germany) with the MagNA Pure 96 DNA and viral NA Small Volume Kit (Roche; Cat ID. 06543588001) and the MagNA Pure 96 System Fluid (Roche; Cat ID. 05467578001).

Template RNA from the viral extract was used for cDNA synthesis using the LightCycler 480 RNA Master Hydrolysis Probes (Roche; Cat ID. 04991885001). The cDNA products were then amplified in a real‐time RT‐PCR procedure carried out by a LightCycler instrument (Roche Applied Science). Each reaction had a total volume of 20 μL containing 5 μL of the viral RNA extract, 1× of RNA Master Hydrolysis Probes, 3.25 mmol/L of Mn(OAc)_2_, 1× of enhancer solution, 0.2 μmol/L of Influenza A/B probes, 0.8 μmol/L of Influenza A/B forward primers, 0.8 μmol/L of Influenza A/B reverse primers, and water. Equine arteritis virus (EAV) was used as an internal control and included in each reaction with 0.04 μmol/L of EAV probes, 0.2 μmol/L of EAV forward primers, and 0.2 μmol/L of EAV reverse primers. Thermal cycling conditions were set up as follow: reverse transcription at 58°C for 20 minutes, enzyme inactivation at 95°C for 5 minutes, and 45 cycles of 95°C for 15 seconds, 55°C for 30 seconds, and 72°C for 20 seconds. Fluorescent signals were measured by LightCycler software, at wavelengths between 465 and 510 nm for influenza A and B.

### 2.3 Climate data

Data on daily mean temperature (*T*) and relative humidity (RH) were collected from Weather Underground for Ho Chi Minh City, Vietnam (http://www.wunderground.com), from the beginning of 2000 until the end of 2015. Absolute humidity (AH) was calculated using relative humidity and temperature:

(1) $$\text{AH}=\frac{6.112\times \exp\left(\frac{17.67\times T}{243.5+T}\right)\times 2.1674\times \text{RH}}{273.15+T}$$

The series of daily climate data were smoothed with a 15‐day moving average before being used in our analyses.

### 2.4 Time series detrending and standardization

A total of 28 regularly reporting clinics (those who reported at least 200 reports from 2010 to 2015 and reported positive ILI numbers at least half of the time) were included in the time series analysis. A 29th clinic that met these inclusion criteria was removed for quality control reasons. The ILI data of 2009 were not used in the analysis due to the small number of reporting clinics during the first 5 months of the study. Each clinic's time series was converted to a *z*‐score scale by computing the *z*‐score of each ILI percentage inside a 12‐month moving window (centered at the calculated data point), thus removing long‐term trends in the data; we verified that window sizes of 6, 9, 15, and 18 months did not have any qualitative effects on the overall ILI trends. The daily *z*‐scores were averaged across clinics and smoothed using a 15‐day window to construct the ILI *z*‐score time series that we used in our subsequent analysis (see Figure S1 for effects of different smoothing windows).

The time series was validated by verifying that it was not white noise (*P*‐value <10^−15^, Box‐Ljung test) and by showing that the majority of individual clinics had a higher correlation to the aggregate time series than would be expected if reporting were random (Figure S2).

### 2.5 Statistical analysis and forecasting

Periodicity and frequency decomposition in the smoothed 6‐year ILI trend were assessed with a standard autocorrelation function (ACF) and a discrete Fourier transform (DFT). The ILI *z*‐score time series was regressed (linear link function) onto linear and nonlinear variants of the climate variables (*T*, RH, AH, √*T*, √RH, √AH, *T* ^2^, RH^2^, and AH^2^) to determine which nonlinear effects were present, as there is some evidence of nonlinear effects of climate on ILI.^67^ In addition, a time‐dependent fixed effect *α*_*j*_ mimicking the dominant periodicity identified by the ACF (here, 206 days) was included on the right‐hand side of the regression equation. Twenty‐one *α*_*j*_ were allowed for in the model, meaning that periodicity in the system is modeled with a piecewise constant function taking 21 different values during a full period of 206 days. This is equivalent to having 21 fixed‐effect terms in a regression, each multiplied by an indicator variable describing whether that data point belongs to that period, ensuring that only one fixed‐effect term is added at a time. The piecewise constant function has an advantage over the sinusoidal approach traditionally used in epidemiological analyses because the stepwise nature of the *α*_*j*_ allows the periodicity in the system to take any shape determined by the data and does not require that the forcing function to be sinusoidal or continuous. In exploring the shape of this function, it was found that more than seven pieces are needed to prevent the model forecasts from appearing too step‐like.

The nonannual cycle, *T*, √RH, and RH were the explanatory terms according to the Akaike information criterion (AIC) using the stepwise regression approach in R (*step*() function). The ILI *z*‐scores were then regressed onto the nonannual cycle, *T*, √RH, and RH, and lagged versions of these climate variables, extending back 5 weeks in the past. The same stepwise regression approach (*step*() function in R) using the AIC was used to remove regression terms that did not add explanatory power. The selected regression equation is

(2) $$z_{i}=\beta_{1}T+\beta_{2}\text{RH}+\beta_{3}\left(T\times \text{RH}\right)+\beta_{4}\sqrt{\text{RH}}+\beta_{5}T_{\mathrm{lag}3}+\beta_{6}T_{\mathrm{lag}4}+\beta_{7}T_{\mathrm{lag}5}+\beta_{8}{\text{RH}}_{\mathrm{lag}5}+\beta_{9}\sqrt{{\text{RH}}_{\mathrm{lag}5}}+\alpha_{j}\sum_{j=1}^{21}1_{\left[\mathrm{day}\;i\;\mathrm{belongs}\;\mathrm{to}\;\mathrm{period}\;j\right]}$$

To determine whether the regression approach offers any predictability in the system, we inferred the regression coefficients and the time‐dependent fixed effects using the first 3 years of data from January 1, 2010 to December 31, 2012, and we compared the predicted and real ILI trends for 2013‐2015. The *median prediction error* was defined simply as the median of the absolute differences between the predicted *z*‐score time series and the real *z*‐score time series. We varied the size of the training set to determine how many years of data would be needed to achieve robustness in predictability (Figure S4).

### 2.6 Bootstrapping climate data

To test the robustness of this prediction to changes in the annual climate cycle and the system's intrinsic (dominant) cycle identified by the ACF (206 days), we removed the annual trend in the climate cycle with a smoothing‐by‐bootstrapping approach and we artificially varied the length *c* of the intrinsic nonannual cycle. To create a bootstrap‐smoothed climate time series, we defined the climate variables for each time point at *t*_bss_ in 2010‐2015 as a random sample taken during 2000‐2015 and within *d* calendar days of *t*_bss_ (see Figure S5). As *d* increases, the annual structure of the climate cycle gradually vanishes. Two hundred bootstrapped time series were created (for each climate variable), for each cycle length *c*, and for each climate subsampling window *d*. For each (*c*,* d*) pair, regression (onto each of the 200 bootstrapped time series separately) and prediction (using each bootstrapped series of 2013‐2015 separately) were re‐performed, and the median prediction error was plotted to determine whether changing assumptions about the length of the intrinsic cycle or the strength/amplitude of the climate data had a detrimental effect on predictability in our system. Mean prediction errors are shown in Figure S7.

All sampling, bootstrapping, and statistical analyses were performed in R (version 3.2.1; Vienna, Austria).

## 3 RESULTS

**Figure 1:**
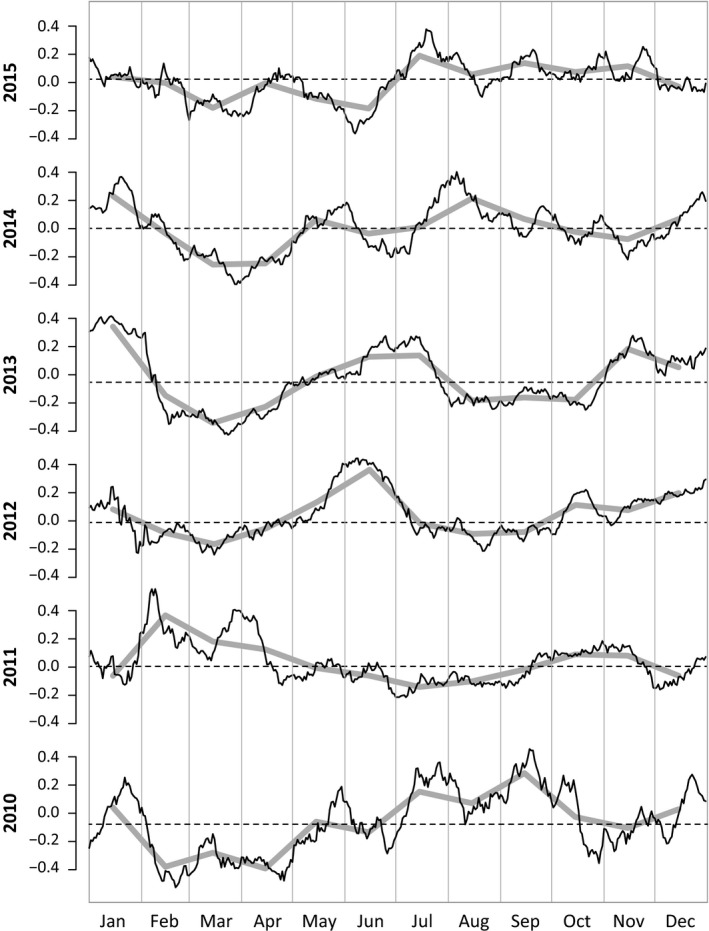
Trends in ILI *z*‐scores by year. The black lines show 15‐d moving‐average smoothed *z*‐scores (after detrending). The gray solid lines show the monthly mean *z*‐score values. The horizontal dashed lines represent the median ILI *z*‐score for that year. ILI, influenza‐like illness

A total of 63 clinics were enrolled during the study, about half of which reported regularly, and 36 920 daily reports were received from August 10, 2009 to December 31, 2015, corresponding to 1 727 076 outpatients and 183 596 outpatients meeting the clinical definition of ILI. The median clinic saw an average of 30 patients per day (IQR: 16‐50 across clinics). Approximately 10.6% of all patients were classified as ILI, and this percentage exhibited a decreasing trend during the first 6 years of the study (Table 1). To create a single ILI time series for Ho Chi Minh City, we detrended and standardized each clinic's ILI percentages to a *z*‐score scale and then aggregated these into a single *z*‐score time series. Several internal validations were carried out to ensure that the data followed certain expected behaviors for multisite syndromic reporting and that arbitrary or random reports were not being sent during the course of the study (see Materials and Methods). In particular, note that individual clinic time series correlated with each other, and replacing a single clinic with a white noise signal of equal variance reduced the correlation between that clinic and the aggregate ILI trend (Figure S2). ILI trends in Ho Chi Minh City (Figure 1) suggest that there are typically multiple ILI peaks per year, as has been observed in other tropical and subtropical regions.^28^, ^30^, ^61^ Visually, no seasonal or annual cycle appears in these data.

**Table 1:** Summary of ILI reports for 2009‐2015

Year	Clinics reporting at least			Total patients	Reported ILI cases	ILI percentage	
	1 d	50 d	150 d			Median	IQR
2009^a^	19	10	0	35 115	10 163	24.40	19.36, 35.89
2010	27	15	7	103 396	24 922	15.42	3.82, 26.89
2011	28	24	20	275 033	35 176	14.73	4.13, 25.86
2012	35	28	25	375 077	42 373	13.30	6.25, 26.49
2013	30	28	23	385 300	30 183	9.99	2.47, 20.61
2014	32	27	20	300 223	19 461	10.64	2.67, 16.14
2015	35	26	21	252 932	21 318	11.69	6.86, 17.58

ILI, influenza‐like illness.

a Data collection in 2009 started on August 10th.

**Figure 2:**
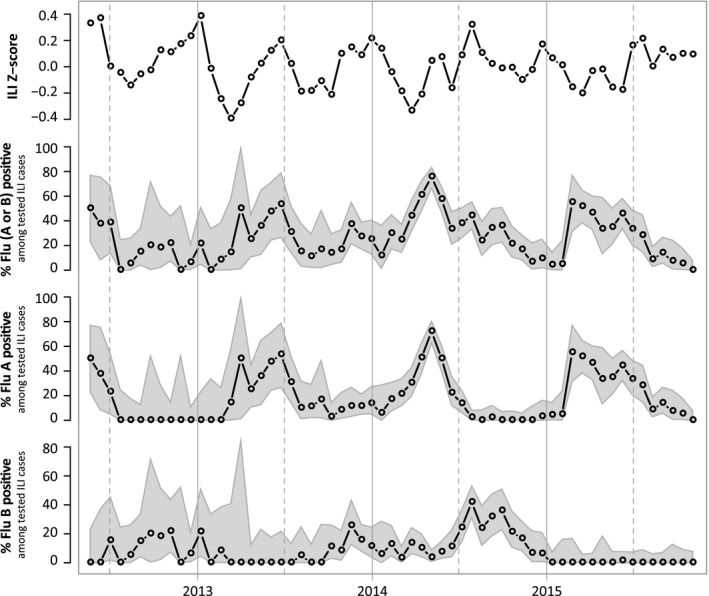
Time series of ILI *z*‐score and influenza PCR‐positivity, in 3‐wk windows, for the period of time when PCR confirmations were being carried out in the clinics in the study. Gray region around flu‐positive percentage is the 95% confidence region computed using the exact binomial method. The Pearson correlation between the time series is shown in TableS2. ILI, influenza‐like illness

In a subset of the clinics, molecular confirmations on nasopharyngeal samples (n* *=* *2217) were taken from May 2012 to December 2015. Compared to other tropical settings, these clinics had a rate of influenza positivity (21.5% positivity for influenza A and 9.7% positivity for influenza B) in the high range of previously published studies.^26^, ^37^, ^42^, ^50^, ^51^, ^68^ We compared the confirmed influenza cases to the ILI data and found that there was no correlation between the two time series (Figure 2; Pearson correlation coefficient: −0.02, *P*‐value: 0.86) and that this did not differ for influenza A and B individually (both *P*‐values >0.15). The time series showed periods of high ILI activity with a low level of influenza confirmation, likely representing epidemic waves of other respiratory viruses, as well as periods that were high influenza and low ILI, suggesting that influenza may not drive the overall trend of ILI incidence as clearly as it does in temperate regions.^69^, ^70^, ^71^, ^72^, ^73^

**Figure 3:**
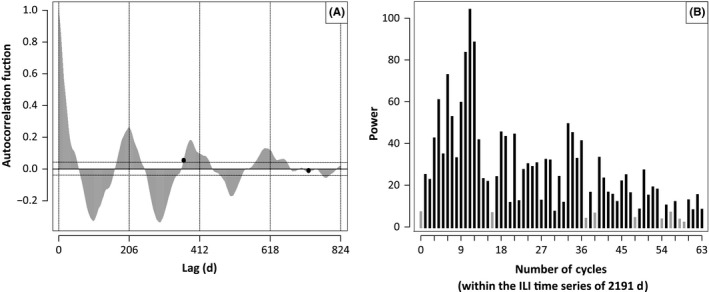
A, Autocorrelation function (ACF) for the *z*‐score time series. Horizontal dashed lines demark the statistically significant regions (*P *<* *0.05). Black dots represent the ACF values of lags of 365 and 730 d. The first peak in the ACF is at the lag of 206 d. B, Discrete Fourier transform (DFT) of the *z*‐score time series. The period length of each DFT can be calculated by dividing 2191 (the number of days in the time series) by the corresponding number of cycles (the frequency of the DFT). Frequencies whose power is lower than 6.93 (ie, periodic functions whose correlation with the *z*‐score time series is lower than their correlation with a constant signal) are shown in gray. The DFT reaches its highest power at 11 cycles, corresponding to a cycle length of 199 d

We identified a dominant periodicity in the data using an ACF and standard time series decomposition (see Materials and Methods). The ACF identified 206 days (ACF = 0.262; *P*‐value <10^−15^), whereas the DFT identified 199 days as the time series’ dominant periodic signal (ACF = 0.244 for a lag of 199 days; *P*‐value <10^−15^); see Figure 3. This nonannual signal is almost twice as strong as the annual signal, with the 365‐day lag exhibiting an autocorrelation value of 0.153 (*P*‐value = 0.014); note that the large number of data points results in statistical significance for nearly all ACF values. A dominant nonannual signal is an unusual feature in disease incidence data. We verified that this result was not an artifact of our data renormalization and detrending methods by applying these same methods to temperate zone ILI data and showing that ILI time series in Europe and North America show their strongest periodic signals at 365 days, with no evidence of periodic signals shorter than 1 year (Figure S3).

To determine the relative influence of annual and nonannual signals on the ILI trend, we performed a stepwise regression of the ILI trend onto both annual climatic variables and the system's intrinsic nonannual cycle. Lagged variables, interactions, and nonlinear transformations of the climate variables were included; the nonannual cycle was constructed as a step function with periodicity 206 days (see Materials and Methods). The stepwise regression indicated that the terms with explanatory power were the daily temperature, relative humidity (RH and √RH), the interaction term between RH and temperature, lagged climate terms, and the nonannual cycle (see Table 2). When factoring in interactions and nonlinear terms, the effects of climate are not very strong. At 75% relative humidity, an increase in 1°C is associated with a 0.085 decrease in ILI on the *z*‐score scale. At 28°C and 75% relative humidity, a 10% increase in relative humidity is associated with a 0.034 increase in the ILI *z*‐score. The association between the nonannual cycle and the ILI trend is statistically significant, and the nonannual effect is identified using the Akaike information criterion as a component of the best fit model. Nevertheless, it is important to remember that the number of data points (~37 000) results in statistical significance for a large number of annual and nonannual covariates. Thus, additional robustness analyses were performed.

**Table 2:** Estimates of coefficients from regressing the smoothed daily ILI *z*‐scores (2010‐2012) onto two climate variables, an interaction term, and the temporal indicator variables that were used to construct a periodic 206‐d forcing function in the time series

Coefficient	Estimate	Standard error	*t* statistic	*P*‐value
Intercept	63.5198	6.1223	10.3751	4.31E‐24
Temperature	−1.0446	0.0719	−14.5345	8.43E‐44
3‐wk lagged temperature	0.0004	0.0128	0.0338	9.73E‐01
4‐wk lagged temperature	−0.0232	0.0180	−1.2894	1.98E‐01
5‐wk lagged temperature	0.0843	0.0120	7.0508	3.19E‐12
Relative humidity	−0.0584	0.0375	−1.5569	1.20E‐01
Square root relative humidity	−5.3047	0.7488	−7.0839	2.54E‐12
5‐wk lagged relative humidity (RH.lag5)	0.1656	0.0412	4.0184	6.27E‐05
Square root RH.lag5	−2.8670	0.7278	−3.9393	8.70E‐05
Relative humidity × Temperature	0.0128	0.0009	13.5127	1.60E‐38
Temporal interval				
2	0.0098	0.0275	0.3576	7.21E‐01
3	−0.0963	0.0274	−3.5128	4.62E‐04
4	−0.1900	0.0275	−6.9097	8.34E‐12
5	−0.2111	0.0275	−7.6857	3.44E‐14
6	−0.2178	0.0282	−7.7159	2.75E‐14
7	−0.1105	0.0274	−4.0262	6.07E‐05
8	−0.1036	0.0279	−3.7205	2.09E‐04
9	−0.1442	0.0275	−5.2471	1.86E‐07
10	−0.0867	0.0278	−3.1233	1.84E‐03
11	−0.1811	0.0293	−6.1726	9.53E‐10
12	−0.1949	0.0280	−6.9524	6.25E‐12
13	−0.2311	0.0279	−8.2917	3.33E‐16
14	−0.0828	0.0274	−3.0191	2.60E‐03
15	0.0290	0.0275	1.0569	2.91E‐01
16	−0.0563	0.0267	−2.1069	3.54E‐02
17	0.0047	0.0262	0.1790	8.58E‐01
18	0.0347	0.0265	1.3107	1.90E‐01
19	0.0072	0.0263	0.2750	7.83E‐01
20	−0.0101	0.0260	−0.3873	6.99E‐01
21	0.0548	0.0268	2.0440	4.12E‐02

ILI, influenza‐like illness.

Temperature was measured in Celsius.

**Figure 4:**
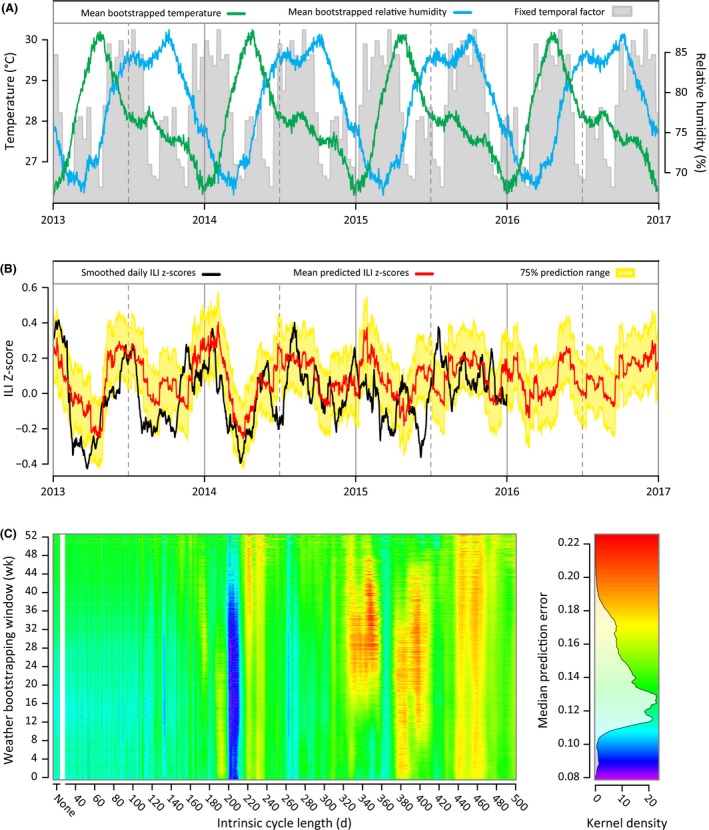
Forecasting ILI *z*‐scores with bootstrapped weather data. A, Annual average temperature trend (green) and relative humidity trend (blue) based on 2000‐2015 weather data for Ho Chi Minh City. Bootstrapping is carried out in a 21‐d window around each time point, which has the effect of smoothing the data with a 21‐d window. The shaded gray area shows the inferred periodic signal from equation (2) using the 2010‐2012 *z*‐scores and assuming a 206‐d cycle. B, Predicted daily ILI *z*‐scores from the regression model (red) and their 75% prediction range (yellow) are plotted alongside with the daily ILI *z*‐scores (black). Model parameters were estimated by regressing ILI *z*‐scores of 2010‐2012 on the real weather data of 2010‐2012. Predictions were calculated based on bootstrapped weather data (see Materials and Methods). The median prediction error from January 1, 2013 to December 31, 2015 is 0.125 (*z*‐score scale, IQR: 0.064, 0.203). C, Median prediction errors when varying both the width of the bootstrapping window *d* for the weather data and the duration of the intrinsic cycle *c* in the system (see Methods). The minimum prediction error is achieved with a weather bootstrapping window of 199 d and an intrinsic cycle of 202 d. ILI, influenza‐like illness

As a third validation of the existence of a nonannual cycle as a true feature of respiratory disease transmission in Ho Chi Minh City, we tested the sensitivity of the ILI forecast accuracy to the length of the nonannual cycle and to the amplitude of the trends of climate variables. The rationale is that if an intrinsic nonannual cycle truly influences respiratory disease dynamics, then (a) forecasting of respiratory disease should be possible using the nonannual cycle, and (b) the forecasts should be less accurate if the nonannual cycle is not used or if an artificial nonannual cycle of a different periodicity is used. Regressing the 2010‐2012 portion of the time series onto the AIC‐selected covariates (including the nonannual cycle of length *c *=* *206), we were able to predict the 2013‐2015 ILI time series with a median absolute error of 0.129 on a *z*‐score scale (Figure S6A). A sensitivity analysis indicated that forecast accuracy is very sensitive to the intrinsic cycle length and that forecast accuracy is reduced substantially if the length *c* of the nonannual cycle is changed by a small amount (Figure 4); the median prediction error is approximately 40%‐50% higher when forecasting is performed with a cycle length *c *<* *195 or *c *>* *215. The increase in prediction error is small or nonexistent when the climate variables are smoothed to reduce their correspondence with the true climate time series (Figure 4). Thus, the nonannual cycle is the key characteristic of this dynamical system that enables accurate forecasting.

Several robustness tests were performed. Figure S6 shows that forecasting using a 202‐day intrinsic nonannual cycle in combination with bootstrapped climate data gives the most accurate forecasts and that a 211‐day cycle was optimal when forecasting ILI trends using real weather data. These results are robust to whether mean or median prediction error is used as an evaluation criterion (Figure S7). Using a simpler regression model with no lags and no nonlinear climate terms, a 201‐day cycle gave the lowest prediction errors (Figures S8 and S9). All analyses provided support for the existence of a nonannual cycle with periodicity of approximately 200 days.

Our decomposition, stepwise regression, and prediction analyses provide strong evidence that an intrinsic nonannual cycle of around 200 days exists for respiratory disease transmission in Ho Chi Minh City. This cycle is either unique to the dynamics of respiratory infections in tropical climates, or it is a natural part of respiratory disease epidemiology in all regions but not detectable in temperate countries as a result of being overwhelmed by the strong winter seasonality of respiratory disease transmission. An ILI indicator, showing whether ILI percentages are above or below the mean trend, is updated daily and publicly available (http://www.ili.vn) providing a real‐time surveillance system for patients and clinical providers.

## 4 DISCUSSION

Our study demonstrates the value of community epidemiology studies for describing fine‐scale dynamics of ILI in tropical settings where respiratory disease dynamics are nonannual and difficult to predict. We were able to show that a network of community clinics can generate a high‐quality syndromic time series that can be used to understand local transmission patterns of respiratory disease and that such a network can generate a significantly larger data set (~6000 data points per year) than traditional surveillance systems that report weekly or monthly measures of incidence. This volume of data increases statistical power to detect ILI associations as well as the presence of nonannual forcing in the system. The present study does not achieve the data volume seen in “big‐data” study designs^1^, ^4^, ^5^, ^74^ which can have tens of millions of observations per year, but the specificity of our data signal is higher than in the aforementioned studies as each data point in our study corresponds to a patient, seen by a physician, determined to have met or not met the clinical criteria for ILI.

The major quality control challenge we encountered was accounting for long‐term trends in ILI (we had a downward trend in our data). In a multisite time series, detrending must be carried out carefully, and changes in a site's reporting patterns must be investigated individually. From discussions with the reporting physicians in our study, the putative causes of the decreasing trend in ILI were likely to have been (a) a more than doubling of patient visit costs that would have reduced the likelihood of reporting a minor respiratory illness, (b) increased clinical specialization at some sites, or (c) more conservative interpretation of ILI guidelines after molecular diagnostics were introduced in May 2012. In addition, during 2011 and 2012, a few large clinics were enrolled in the study, and some of these had higher patient volumes but lower ILI percentages. All of these features of community‐based syndromic reporting systems need to be considered for both study design and surveillance purposes. Detrending with a 12‐month moving average appears to be the simplest way to detrend and preserve any potential annual structure in the data.

The lack of correlation between influenza trends and ILI trends suggests that the transmission dynamics of respiratory disease differ between tropical and temperate zones, consistent with the past decade's literature on this topic.^24^, ^27^, ^28^, ^30^, ^60^, ^63^ Given the observed pattern of multiple ILI peaks in our data, some of which are influenza epidemics and some of which are not, the natural hypothesis explaining this pattern is that multiple respiratory pathogens cocirculate and cause asynchronous epidemics. It is unknown whether in such a system multiple respiratory pathogens should circulate independently or not. The putative mechanism that would create dependence or interference among waves of different cocirculating respiratory viruses would be postinfection raised antibody or cytokine concentrations^75^, ^76^, ^77^ generated by one viral epidemic preventing an epidemic of a different virus from taking off immediately thereafter. Epidemiological interference among respiratory viruses has been observed in long‐term time series in temperate^78^, ^79^ and tropical^80^ regions, but there is still little direct evidence showing that near‐term postinfection immune responses to one respiratory pathogen can affect the outbreak potential of another respiratory pathogen. In our community study, additional molecular confirmations for a range of respiratory pathogens are now underway to further describe this phenomenon.

The second major question that arises from the basic correlational analysis between ILI and influenza is why high influenza periods should be observed when ILI is low. To the best of our knowledge, this pattern has not been observed in other surveillance systems, as a wave of influenza infections is normally sufficient to generate a substantial uptick in the ILI signal. The likely explanation for a high‐influenza low‐ILI period is a larger than expected prevalence of other respiratory viruses among the reported ILI cases; this is possible as the community clinics in our study are almost exclusively outpatient and likely to see many mild cases of respiratory disease. If influenza infection represents only a small fraction of respiratory disease among these outpatients, a wave of influenza alone would not generate an ILI peak. In general, community‐based studies of respiratory disease should aim to characterize the contribution of all respiratory viruses to the ILI trend to determine whether it is a particular pathogen's dominance or synchrony among certain pathogens that generates an ILI peak.

The major finding in our study is that the dominant periodicity observed in our ILI time series is nonannual. This is the first report of a nonannual disease cycle in temperate or tropical respiratory disease data. The existence of an intrinsic nonannual cycle in the dynamics is supported by traditional time series decomposition, by a regression of the time series onto both annual and nonannual covariates, and by an analysis of the system's predictability showing that accurate forecasts of ILI trends are highly dependent on the system's nonannual cycle of ~200 days. The presence of nonannual periodicity is consistent with a mechanism of postinfection immunity conferred by one respiratory virus that affords near‐term protection (3‐6 months) against infection with other respiratory viruses. Data on the rate of antibody decay after acute influenza infection are consistent with this hypothesis,^75^, ^76^ but unfortunately, no such data exist for other respiratory viruses. If the short‐term immunity hypothesis can be shown to be true, then immunological interference among viruses may be the fundamental driver of the immuno‐epidemiology of respiratory disease transmission in the tropics. In temperate countries, where strong wintertime seasonality synchronizes respiratory disease transmission, the interference hypothesis may not be testable due to the short transmission season. In the tropics, where there is no winter to structure the dynamics of respiratory virus transmission, individual viral epidemics may create postepidemic niches—unfavorable to other respiratory pathogens—by generating temporary waves of immunity.

Although a complete forecasting evaluation will require a separate analysis, we can already detect one clear limitation of ILI forecasting methods: that they must be based on future weather predictions which, in our analysis, were bootstrapped from past weather data. Nevertheless, this proved to be a small obstacle in our analysis as, for Ho Chi Minh City, the bootstrapped climate variables yielded accurate predictions of averages for temperature and relative humidity (Figure S5). In other words, it is more likely that higher levels of ILI during a particular period are affected by the average climate behavior during that period and not by any particular days that have extremes in temperature or relative humidity. This contrasts with the climate mechanisms proposed in temperate zones where it is postulated that the onset of abnormally low absolute humidity is closely associated with the onset of the influenza season.^57^ The larger question on climate effects and influenza—why AH, RH, and temperature appear to have different transmission effects in temperate and tropical regions^28^, ^60^, ^81^—remains to be answered. In addition, the lagged effects found in our study (for temperature and relative humidity) should be investigated in other locations to determine whether a period with particular climatic features can result in an increase or decrease in viral transmission that is detected by larger case numbers several weeks later. Much work remains to be done before respiratory disease outbreaks in the tropics can be forecast accurately; our hope is that the nonannual signal identified in this study will help in this endeavor.

A second limitation in the current study design is the lack of age information. We experimented with several different reporting methods (SMS, email, log books) for this study, but only the logbook method was able to capture age information consistently. Unfortunately, this method was adopted by a minority of the clinics in our study, and it was not compatible with real‐time reporting. The age distribution of ILI cases represents a critical data gap in our study and in other mHealth studies that aim at real‐time reporting, as the age distribution could tell us whether the major disease burden skews toward childhood respiratory diseases or general respiratory diseases like influenza. As tropical countries have younger age distributions than temperate countries, this difference may have a profound epidemiological effect on differences in ILI dynamics between temperate and tropical zones, as well as the proportion of ILI cases that are caused by influenza vs other respiratory viruses.

The public health value of our mHealth reporting system is that ILI results can be fed back in real time to participating physicians and the community of health professionals in Ho Chi Minh City. Real‐time ILI trends from our study are publicly available and updated daily. The two key questions raised by our study are (a) to what extent the transmission of noninfluenza respiratory viruses in the tropics is a potential driver of complex multipathogen transmission system and (b) whether it is useful to attempt the timing of influenza vaccination in an epidemiological scenario where influenza epidemics occur irregularly. We aim to investigate the first of these questions by introducing more respiratory virus diagnostics into our study. The second question can be evaluated with a mathematical model of influenza epidemiology, but will necessitate a longer influenza time series and a better understanding of the key drivers of influenza virus dynamics in tropical settings.

## CONFLICT OF INTEREST

MFB has acted as a consultant to Visterra Inc in Cambridge, MA.

## AUTHOR CONTRIBUTIONS

MFB and JF designed the study. HML and AW analyzed the data. HML designed and implemented the real‐time surveillance website http://www.ili.vn. NTH and NTLT recruited participating clinicians and managed study operations and communication. TDN designed the database and text‐parsing tools. NTDN was responsible for management and quality control of the data. ST and DNV helped in data analysis and interpretation of results. NTH, NHTV, and TTNT performed molecular diagnostics. PTT, NNQM, JEB, COB, TVN, NVVC, GET, DTHT, and HV helped interpret the data, especially variability in reporting trends. PTT, DTHT, and HV led study reevaluation at various points to ensure high enrollment and improve data quality. MFB, HML, and AW wrote the first draft of the manuscript.
