# Supplementary material for: Nonannual seasonality of influenza‐like illness in a tropical urban setting
Source: Influenza Other Respir Viruses. 2018 Aug 21;12(6):742–54. doi: 10.1111/irv.12595 (PMC6185894; doi:10.1111/irv.12595)
Supplement: Supplementary file 1 [file IRV-12-742-s001.docx]

**Table S1:** Influenza Primers and Probes

| Influenza A Forward | 5’-GAC CRA TCC TGT CAC CTC TGA C-3’ |
| --- | --- |
| Influenza A Reverse | 5’-AGG GCA TTY TGG ACA AAK CGT CTA-3’ |
| Influenza A Probe | 5’-TGC AGT CCT CGC TCA CTG GGC ACG-3’ |
| Influenza B Forward | 5’-TCC TCA ACT CAC TCT TCG AGC G-3’ |
| Influenza B Reverse | 5’-CGG TGC TCT TGA CCA AAT TGG-3’ |
| Influenza B Probe | 5’-CCA ATT CGA GCA GCT GAA ACT GCG GTG-3’ |

**Table S2:** Pairwise Pearson’s correlation between the time series in Figure 2

|  | Flu A Positivity | Flu B Positivity | ILI Z-score |
| --- | --- | --- | --- |
| All Flu (A & B) Positivity | 0.8519 (p-value < 1e-15) | 0.1185 (p-value = 0.3630) | -0.0236 (p-value = 0.8567) |
| Flu A Positivity |  | -0.4190 (p-value = 0.0008) | -0.1181 (p-value = 0.3648) |
| Flu B Positivity |  |  | 0.1830 (p-value = 0.1582) |

**Figure S1:** The average daily z-scores aggregated from the detrended data of the 28 selected clinics (see Materials & Methods). The unsmoothed aggregated time series is shown in grey lines. The green, blue, and black lines represent the smoothed data using moving windows of sizes 7 days, 15 days, and 21 days respectively.

**Figure S2:** The simulated and real Pearson’s correlations between the detrended data of individual clinics and the aggregated time series. The open and closed black dots show the real Pearson correlation coefficient between an individual clinic and the aggregate trend of the other 27 clinics. To test if these correlation coefficients were larger than what would be observed at random, we replaced each individual clinic by white noise (preserving its mean and variance) and recomputed its correlation to the trend defined by the other 27 clinics. One thousand white noise signals were generated for each clinic, and the box-and-whiskers plots for these 1000 white-noise signals show the 95% range (box) and full range (whiskers) for the simulated Pearson correlation coefficients. Open circles show correlations within the 95% range generated by a white-noise signal, and filled circles are shown for correlations that fall outside the 95% range generated by a white-noise signal.

**Figure S3:** Autocorrelation function (ACF) for temperate zone ILI data that were detrended and standardized to a z-score scale with the same methods used in this paper. US data are from 1997-2015 and are broken up by the ten Health and Human Services (HHS) regions used by the Centers for Disease Control’s ILINet (<http://www.cdc.gov/flu/weekly/overview.htm>). Data from France are from 1984-2011 and come from the general-practitioner network known as Reseau Sentinelles (<http://websenti.u707.jussieu.fr/sentiweb/?page=accueil>). Data from the Netherlands (Jan 2010 – Dec 2014), Belgium (Jan 2007 – Jun 2012), Portugal (Oct 2008 – June 2012), and the United Kingdom (Oct 2008 – Jun 2012) come from the self-reporting of influenza symptoms through the European Network InfluenzaNet (<https://www.influenzanet.eu>). The Portugal and UK data do not have an ACF peak at 365 days, but both show a peak at 730 days. This is possibly the result of the short time series available for these two countries, and the summer 2009 influenza pandemic interrupting normal patterns of influenza transmission.


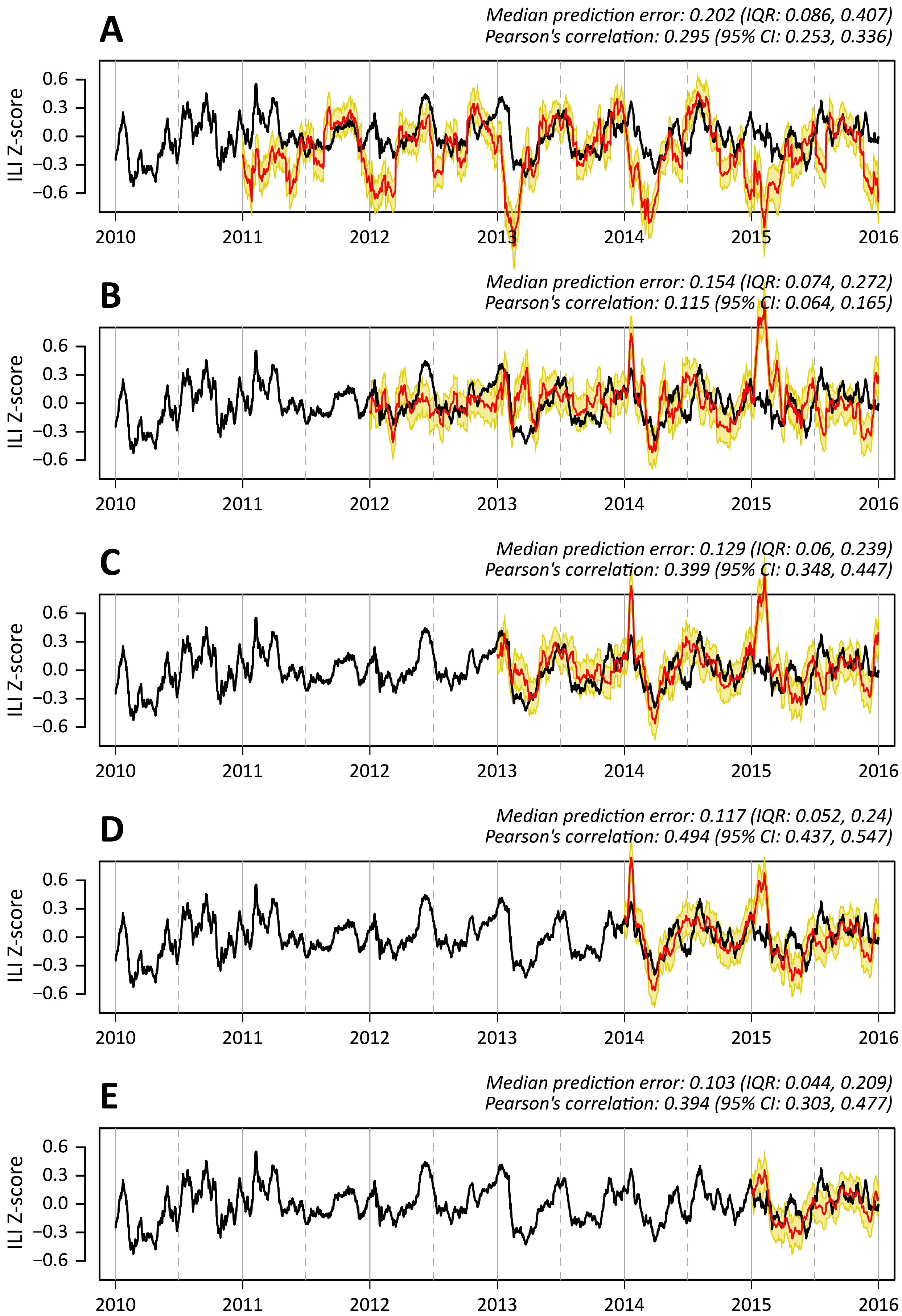


**Figure S4.** ILI forecasts (red) with 75% prediction ranges (yellow) based on inferring the weather and intrinsic cycle coefficients using ILI z-score data through the end of (**A**) 2010, (**B**) 2011, (**C**) 2012, (**D**) 2013, and (**E**) 2014. The ILI z-score data set is shown in black. Median values of prediction errors and Pearson’s correlations (between prediction results and the real data) are shown above each panel. One year of data does not seem to be sufficient to determine the relationship between ILI, climate variables, and the intrinsic cycle in the system.

 

**Figure S5:** The observed daily data and the mean values of 200 bootstraps of (**A**) temperature and (**B**) relative humidity in Ho Chi Minh City from 1^st^ January 2010 to 31^st^ December 2015. Daily bootstrapped data were generated based on real weather data collected from 2000 to the end of 2015 (see Materials & Methods).


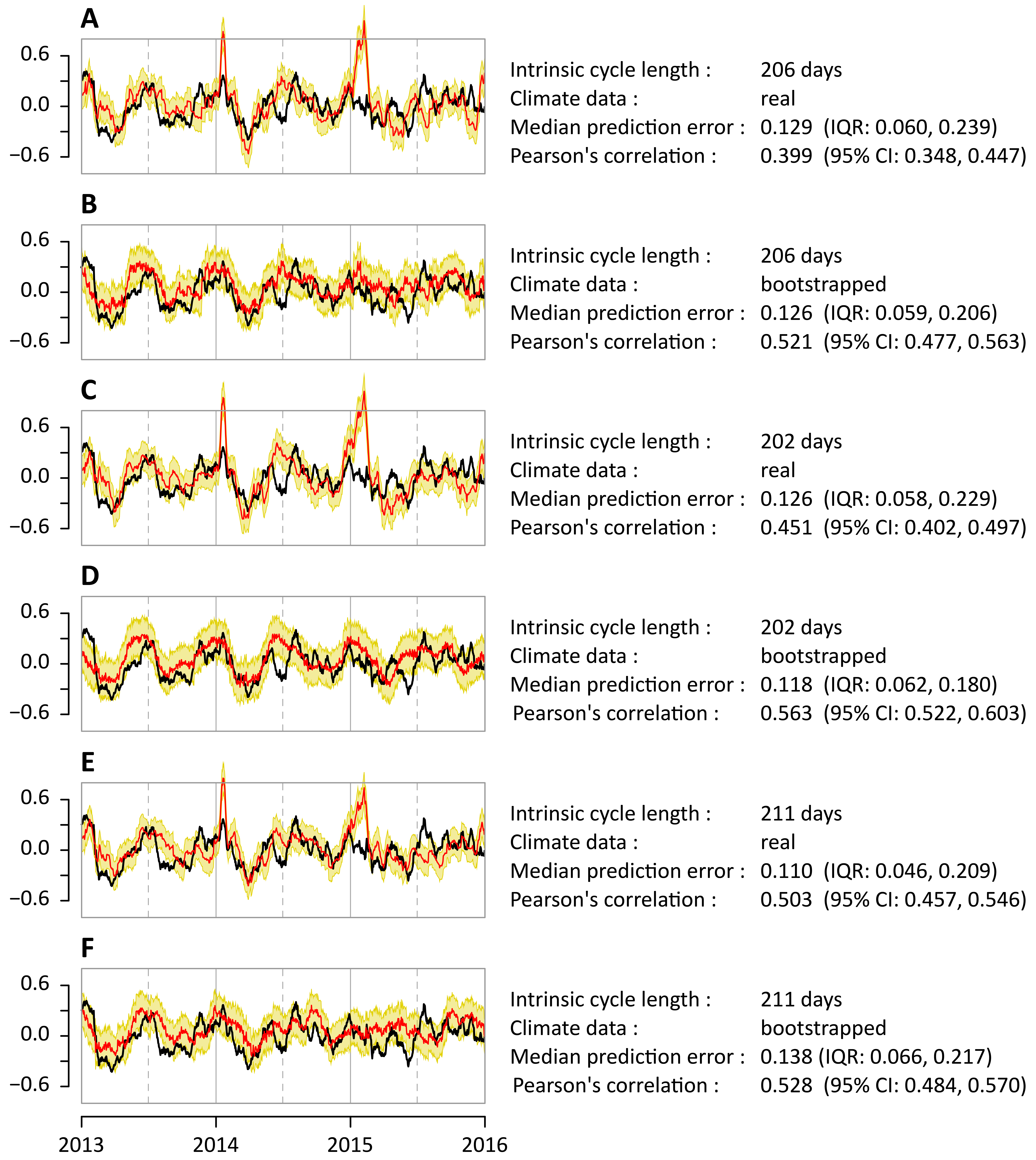


**Figure S6:** ILI forecasts (red) of 2013-2015 with 75% prediction ranges (yellow) based on inferring the weather and intrinsic cycle coefficients using ILI z-score data through the end of 2012. The real ILI z-score data of 2013-2015 is shown in black. The length of the intrinsic cycle was fixed at (**A, B**) 206 days, (**C, D**) 202 days, or (**E, F**) 211 days. Both training and prediction use either real (**A, C, E**) or bootstrapped (**B, D, F**) climate data (temperature & relative humidity). Median values of prediction errors and Pearson’s correlations (between prediction results and the real data) are shown next to each panel. Intrinsic cycle lengths of 190 to 220 were tested out for this analysis. The 202-day cycle had the lowest median prediction error and highest correlation when the bootstrapped climate data were used. Meanwhile, the 211-day cycle gives the best performance when the prediction relied on the real weather data.

**Figure S7:** (**A**) Mean prediction errors when varying the intrinsic cycle length and the bootstrapping window when using bootstrapped weather data. Heat map generated as Figure 4C of main text, but with mean rather than median predictions errors. (**B**) Magnification of panel A (note different color bar). (**C**) Magnification of Figure 4C in main text (note different color bar).

**Linear model with no lagged covariates**

On the next three pages, Figures S8 and S9 show the analysis from Figure 4 and Figure S7, redone with a linear model with no lags. In this simple regression model, only AH, RH, and temperature were included as covariates in the analysis, with no nonlinear terms and no lagged climate terms. AH was removed from the analysis during the stepwise regression procedure. The simpler model gives largely similar results, showing that forecasting is most accurate when the intrinsic cycle length is set between 200 and 205 days. There were no qualitative differences in the forecasting between the linear model and the non-linear lagged model.

**Figure S8.** Forecasting ILI z-scores with bootstrapped weather data, using a linear statistical model with no lags. (**A**) Annual average temperature trend (green) and relative humidity trend (blue) based on 2000-2015 weather data for Ho Chi Minh City. Bootstrapping is done in a 21-day window around each time point, which has the effect of smoothing the data with a 21-day window. The shaded gray area shows the inferred periodic signal from equation (2) using the 2010 to 2012 z-scores and assuming a 206-day cycle. (**B**) Predicted daily ILI z-scores from the regression model (red) and their 75% prediction range (yellow) are plotted alongside with the daily ILI z-scores (black). Model parameters were estimated by regressing ILI z-scores of 2010-2012 on the real weather data of 2010-2012. Predictions were calculated based on bootstrapped weather data. The median prediction error from January 1 2013 to December 31 2015 is 0.107 (z-score scale, IQR: 0.049, 0.186). (**C**) Median prediction errors when varying both the width of the bootstrapping window *d* for the weather data and the duration of the intrinsic cycle *c* in the system. The minimum prediction error is achieved with a weather bootstrapping window of 42 days and an intrinsic cycle of 201 days.

**Figure S9:** Forecasting ILI z-scores with bootstrapped weather data, using a linear statistical model with no lags. (**A**) Mean prediction errors when varying the intrinsic cycle length and the bootstrapping window when using bootstrapped weather data. Heat map generated using mean rather than median predictions errors. (**B**) Magnification of panel A (note different color bar). (**C**) Magnification of Figure S8, panel C (note different color bar).
